# Early Diagnosis and Diagnostic Delay in Oral Cancer

**DOI:** 10.3390/cancers14071758

**Published:** 2022-03-30

**Authors:** Pablo Varela-Centelles

**Affiliations:** C.S. Praza do Ferrol Lugo, A Mariña, Monforte de Lemos Health Area, Galician Health Service, Praza Ferrol 11, 27001 Lugo, Spain; pabloignacio.varela@usc.es

Oral cancer is a very serious public health problem in many parts of the world, particularly in developing countries, where about two-thirds of incident cases occur [1]. There are wide geographical variations in incidence (differences in rates of about 20-fold), with specific regions being especially affected within each continent (e.g., India, Pakistan, and Sri Lanka in Asia; Hungary, Slovakia, and Slovenia in Europe; Brazil and Puerto Rico in America; and Papua New Guinea in the Pacific Region) [2].

A relatively common finding in the literature is that about one-half of oral cancers are diagnosed at advanced stages (III–IV) [3,4], despite the oral cavity being easily accessible for inspection.

Unfortunately, the negative effect that disease-stage at diagnosis has on survival has not been balanced out by the important therapeutic advances witnessed in recent decades, because no major improvements in overall survival to this neoplasm can be observed. Therefore, a paramount question should be what can be done to favor diagnosis at earlier stages: adequate answers to this issue may have important impacts on survival rates. However, this is a complex problem with many facets (socioeconomic, biological, medical, cultural, political, administrative, etc.). Some are common to other cancers, but others are quite specific, and we may be able to take a few advantages from them. A clear example is the possibility for self-exploration. Regrettably, effective self-exploration of the oral cavity requires some sort of elementary training and a certain level of awareness of the disease among risk groups, which is not always feasible [5].

Patient awareness is an important factor in the chain of events resulting in consultation with a healthcare professional, and it has been demonstrated to have potential for modulating the length of time elapsed since the first symptom to the final oral cancer diagnosis. In fact, the chances of people taking longer to present with an advanced-stage tumor at diagnosis are significantly higher than those presenting earlier [6]. In actual fact, the time period exclusively depends on the patient’s behavior, and attitude represents the major component of waiting times until diagnosis, with an average of 80.3 days [7].

The diagnostic interval (professional delay), although shorter (47.9 days), is also an interesting and perhaps more workable target on which efforts for diminishing diagnostic delays should focus.

Thus, strategies to achieve early diagnoses and treatment may have a significant impact not only on patient survival but also on the morbidity and economic burden of the disease. These strategies should consider every factor in the processes of diagnosis and treatment, namely, patient awareness, pathways to primary and definitive diagnoses, diagnostic techniques and treatment circuits, and time intervals in the pathways from first symptom to final treatment.

This Special Issue analyzes the factors, techniques, and agents which disclose areas where improvement is more feasible. In this vein, the paper by Saka-Herrán et al. [8] reviews the main causes influencing time-intervals in the pathway to oral cancer diagnosis from a standardized standpoint and discloses potential targets for research on this topic, focusing on how the pre-treatment and treatment intervals can influence on patient survival.

When dealing with the early diagnosis of oral cancer, special attention should be paid to precancerous lesions, because about 11.3% of them undergo malignant transformations, including the transformation of 42.8% of severe dysplasias into carcinoma [9]. Efforts are being made to disclose those potentially malignant oral disorders more prone to malignant transformations [10,11]. Among them, proliferative verrucous leukoplakia requires specific attention because it presents the highest tendency for recurrence and malignant transformation [12]. The paper by Palaia et al. [13] reports a proportion of 45.8% of malignant transformation for these lesions, mostly to conventional squamous cell carcinoma (OSCC) (3.8-fold) or to verrucous carcinoma. According to González-Moles et al. [12], this high proportion of malignant transformation may have something to do with inconsistencies in its diagnosis; therefore, the real percentage could in fact be higher. This problem is tackled in their contribution to this Special Issue, which maps the existing evidence and explores evidence-based diagnostic criteria to offer a series of aspects which should be complied with in order to reach the maximum level of diagnostic certainty.

The same authors, in another contribution [14], explore the prognosis of these carcinomas developed in patients with proliferative verrucous leukoplakia and concluded that these neoplasms show a favorable prognostic behavior hypothetically related to characteristics inherent to the tumor’s own biopathology.

Beyond any doubt, delays in the diagnosis of oral cancers would be diminished by the identification of specific diagnostic markers. The quest for these molecules is intense, from a range of approaches [15,16], with interesting potential candidates. In the same line, tissue markers exposing early phases of malignant transformation can also contribute to this goal. One of these potential markers may well be ZNF-281, a zinc finger factor involved in cancer progression and metastasis. The article by Starzynska et al. [17] first investigates its role in oral squamous cell carcinoma to reveal that, despite its lack of influence on survival and poor prognostic importance and its imprecise value as a marker for the clinical and pathological development of cancer, the ZNF-281 H-score levels seem to be able to differentiate normal tissue from OSCC. This ability makes ZNF-281 an interesting potential coadjutant for the diagnosis of poorly differentiated OSCC and in the assessment of tumor recurrence after radiotherapy.

Oral cancer diagnosis will also benefit from the introduction of digital medicine. In particular, artificial intelligence tools using non-invasive techniques offer excellent opportunities, which are thoroughly reviewed in the paper by García-Pola et al. [18].

Unlike other neoplasms, oral cancer can be diagnosed by two very different professional profiles (physicians and dentists) in distinct settings. This circumstance may condition not only the referral pathways, but also the trigger consultations with each profile. The article by Varela-Centelles et al. [19] investigates the impacts of the type of the first symptom of oral cancer on the time intervals to diagnosis. This study shows that, despite specific training opportunities [20], dentists generate longer primary care intervals and need a higher number of consultations than physicians before deciding to refer an oral cancer patient. Conversely, physicians consistently use less efficient in-hospital routes, resulting in longer total intervals.

This Special Issue offers an overall perspective of the problem of the diagnostic delay of oral cancer and discloses targets for future research with potential translational effects in terms of reduced patient morbidity, improved survival rates, and alleviated financial burdens for healthcare systems.
